# You shall know an object by the company it keeps: An investigation of semantic representations derived from object co-occurrence in visual scenes

**DOI:** 10.1016/j.neuropsychologia.2014.08.031

**Published:** 2015-09

**Authors:** Zahra Sadeghi, James L. McClelland, Paul Hoffman

**Affiliations:** a School of Electrical and Computer Engineering, University of Tehran, Iran; b Department of Psychology, Center for Mind, Brain and Computation, Stanford University, Stanford, CA, USA; c Neuroscience and Aphasia Research Unit (NARU), School of Psychological Sciences, University of Manchester, Zochonis Building, Oxford Road, Manchester M13 9PL, UK

**Keywords:** Semantic representation, Object knowledge, Latent semantic analysis, Categorisation

## Abstract

An influential position in lexical semantics holds that semantic representations for words can be derived through analysis of patterns of lexical co-occurrence in large language corpora. Firth (1957) famously summarised this principle as “you shall know a word by the company it keeps”. We explored whether the same principle could be applied to non-verbal patterns of object co-occurrence in natural scenes. We performed latent semantic analysis (LSA) on a set of photographed scenes in which all of the objects present had been manually labelled. This resulted in a representation of objects in a high-dimensional space in which similarity between two objects indicated the degree to which they appeared in similar scenes. These representations revealed similarities among objects belonging to the same taxonomic category (e.g., items of clothing) as well as cross-category associations (e.g., between fruits and kitchen utensils). We also compared representations generated from this scene dataset with two established methods for elucidating semantic representations: (a) a published database of semantic features generated verbally by participants and (b) LSA applied to a linguistic corpus in the usual fashion. Statistical comparisons of the three methods indicated significant association between the structures revealed by each method, with the scene dataset displaying greater convergence with feature-based representations than did LSA applied to linguistic data. The results indicate that information about the conceptual significance of objects can be extracted from their patterns of co-occurrence in natural environments, opening the possibility for such data to be incorporated into existing models of conceptual representation.

## Highlights

• Novel application of latent semantic analysis to object co-occurrence data.
• Analysis performed on a corpus of labelled objects in natural visual scenes.
• Category co-ordinates appear in similar scenes, as do semantically associated items.
• Results complement those from standard linguistic LSA and feature listing databases.

## 1 Introduction

The structure and content of the conceptual representations of objects are central topics in the study of semantic cognition. It is widely accepted that our understanding of objects and their relationships with one another can be usefully captured by analysing the properties they possess, often referred to as semantic features. A number of large-scale feature listing studies have been conducted, in which participants are asked to generate features for a large set of objects (Cree & McRae, 2003; Devlin, Gonnerman, Andersen, & Seidenberg, 1998; Garrard, Lambon Ralph, Hodges, & Patterson, 2001; Tyler, Moss, Durrant-Peatfield, & Levy, 2000; Vinson, Vigliocco, Cappa, & Siri, 2003; Zannino, Perri, Pasqualetti, Caltagirone, & Carlesimo, 2006). In such studies, participants tend to produce features derived from perceptual experience (e.g., lemons are yellow), functional features concerned with behaviours or goals associated with the object (lemons are used to make drinks) and more abstract information that can typically only be expressed verbally (lemons are a type of citrus fruit). On this view, two objects are conceptually related to the extent that they share similar features; so oranges are semantically linked with lemons because they too are citrus fruits and are used to make drinks. Feature generation studies of this kind have strongly endorsed the view that object knowledge is organised in terms of taxonomic category. Objects that belong to the same taxonomic category tend to share features (Cree & McRae, 2003) and, moreover, items that share many features with other items from their category are judged to be more prototypical members of the category (Garrard et al., 2001). Dilkina and Lambon Ralph (2012) recently demonstrated that items within the same category most frequently shared features that referred to their perceptual qualities, though functional and more abstract encyclopaedic features were also somewhat linked to taxonomic organisation. The patterning of correlations amongst features and the relative salience of different types of feature have also been shown to vary across living and non-living things (Farah & McClelland, 1991; Garrard et al., 2001; Tyler et al., 2000). Living things are more strongly associated with perceptual features, for example, and manufactured artefacts with functional features. These differences have been proposed to account for patterns of category-selective semantic deficits sometimes observed in a variety of neurological conditions (Cree & McRae, 2003; Farah & McClelland, 1991; Warrington & Shallice, 1984).

The feature-based approach to object knowledge has proved fruitful, with a number of models of object knowledge assuming that object concepts are structured in terms of their featural similarity (Collins & Quillian, 1969; McRae, deSa, & Seidenberg, 1997; Rogers et al., 2004; Rogers & McClelland, 2004; Tyler et al., 2000; Vigliocco, Vinson, Lewis, & Garrett, 2004). The idea that taxonomic category is a key organising principle for object concepts has also guided recent neuroimaging studies that have used multi-voxel pattern analysis to investigate representational structure (Devereux, Clarke, Marouchos, & Tyler, 2013; Fairhall & Caramazza, 2013; Kriegeskorte et al., 2008; Peelen & Caramazza, 2012). Some limitations of the feature-based approach have been noted, however. It has been suggested that the feature generation task is biased towards features that distinguish objects from their category neighbours and towards aspects of information that can be easily expressed verbally (Hoffman & Lambon Ralph, 2013; Rogers et al., 2004). Another, perhaps more fundamental, limitation is the fact that participants generating semantic features are asked to consider each object in isolation. The relationships between objects are therefore inferred indirectly, in terms of their feature overlap. This is not representative of our natural experience with objects. Environments typically contain many objects and most activities require us to interact with multiple objects simultaneously, which often have few features in common. To extend our earlier example, in order to make lemonade, life must give you not only lemons but water, sugar and a jug. How does the co-occurrence of these objects influence our conceptual representations of each of them?

An alternative approach to semantic representation has developed in the field of computational linguistics, based on the idea that semantic representations of words can be derived through statistical analysis of their distribution in large text corpora (Firth, 1957; Griffiths, Steyvers, & Tenenbaum, 2007; Landauer & Dumais, 1997; Lund & Burgess, 1996; Rohde, Gonnerman, & Plaut, 2006). The central tenet underpinning the distributional approach is the idea that words that occur in similar linguistic contexts are related in meaning. On this view, oranges and lemons would be considered similar because they co-occur with a similar set of words in natural language. For example, we might expect both *orange* and *lemon* to frequently occur in sentences that contain words like *squeeze, cut, peel, pips, juice* and *marmalade*. On the face of it, this does not sound so different to the featural approach. However, the distributional approach allows for the possibility that objects from different taxonomic categories which share few features may nevertheless share a semantic relationship (e.g., *lemon* and *ice* may be considered semantically related because both words are used when we talk about making drinks). These associative or thematic relationships are known to play an important role in lexical-semantic processing. For example, significant semantic priming effects occur for word pairs that share an associative relationship as well as items that share semantic features (Alario, Segui, & Ferrand, 2000; Perea & Gotor, 1997; Seidenberg, Waters, Sanders, & Langer, 1984). Furthermore, children readily group objects according to their associative relationships and may even prefer this to grouping by taxonomic similarity (Kagan, Moss, & Sigel, 1963; Smiley & Brown, 1979), suggesting that associations play an important role in the development of concepts. Therefore lexical co-occurrence likely serves as an additional source of constraint over the structuring of object concepts, since it is able to capture associative relationships between items that share few features. However, semantic models based on the distributional principle have been criticised because they rely solely on linguistic data and therefore do not take into account, at least in any direct way, the sensory-motor information available when we perceive and interact with objects in the real world (Andrews, Vigliocco, & Vinson, 2009; Glenberg & Robertson, 2000). Linguistic corpora may code perceptual experiences indirectly, of course, through verbal descriptions of sensory experiences.

**Fig. 1:**
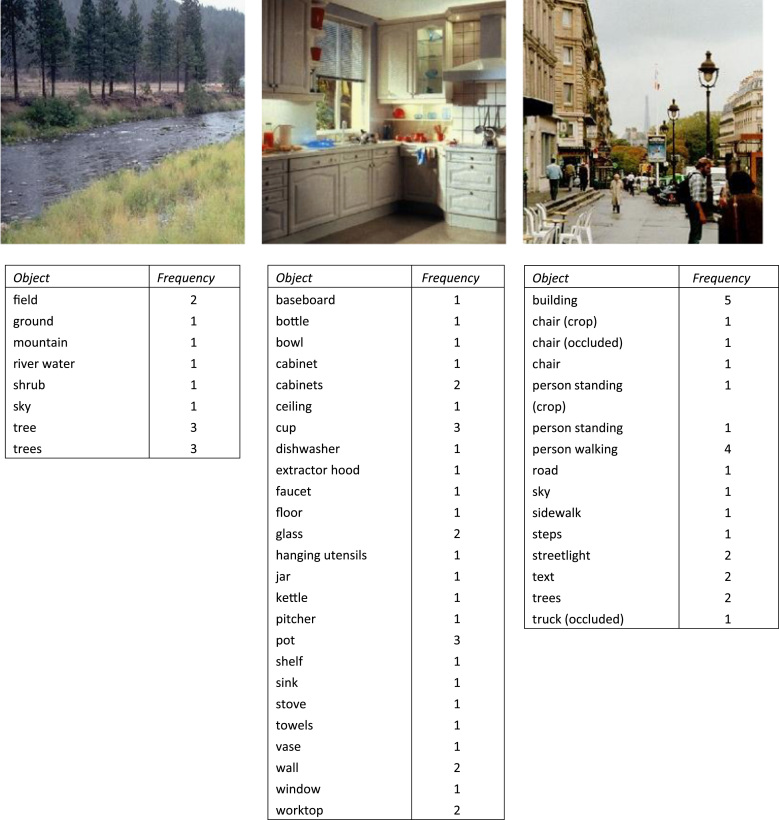
Examples of three images and their object lists from the SUN database.

Feature lists and lexical co-occurrence provide two differing perspectives on the conceptual relationships among objects. There is now evidence that true semantic representation requires a combination of these two sources of data. In an innovative study, Andrews et al. (2009) used a Bayesian probabilistic model to generate semantic representations for objects based jointly on feature lists and word co-occurrence information obtained from a text corpus. The resultant representations provided a better fit to a range of empirical data than those derived from either data source in isolation. This suggests that our understanding of the relationships between objects is based partly on shared properties and partly on knowledge of their co-occurrence. Other researchers have used related statistical methods to integrate feature knowledge with data about concept co-occurrence (Durda, Buchanan, & Caron, 2009; Johns & Jones, 2012; Steyvers, 2010). All of these studies have used linguistic corpus data as the basis for inferring patterns of contextual co-occurrence among objects. However, much of our experience of concrete objects is non-verbal: in addition to using words that refer to objects together in sentences, we also perceive combinations of objects directly in different environments. For example, we frequently see oranges and lemons together in fruit bowls. This direct experience of object co-occurrence potentially provides a rich additional source of information about object concepts, beyond that provided by feature lists and lexical co-occurrence; however, its potential contribution to semantic knowledge has not been assessed. In this study, we investigated whether meaningful semantic information can be derived from patterns of object co-occurrence, by applying latent semantic analysis (LSA) to a set of labelled photographs that depict collections of objects in a variety of natural scenes (see Fig. 1 for examples). LSA is commonly used to derive high-dimensional semantic representations for words based on underlying similarities in the verbal contexts in which they are used (Landauer & Dumais, 1997). Here, we used the same technique to derive high-dimensional semantic representations for objects based on underlying similarities in the environments in which they appear. We compared semantic representations derived in this way with (a) representations based on feature lists (McRae, Cree, Seidenberg, & McNorgan, 2005) and (b) representations obtained through the traditional application of LSA to linguistic corpus data. We aimed to explore the degree to which information derived from environmental co-occurrence provided similar or complementary information about objects as these other two sources.

## 2 Method

### 2.1 Processing of the scene dataset

We used latent semantic analysis (LSA; Landauer & Dumais, 1997) to investigate patterns in visual object co-occurrence. LSA is a well-known technique for constructing semantic representations based on lexical co-occurrence in text corpora. It is typically applied to a corpus of text documents obtained from a variety of sources. A list of the words occurring in the corpus is compiled and the frequency with which each word appears in each document is computed. The result is a highly sparse matrix in which each word is represented as a vector of values that represent the number of times it appears in each of the documents in the corpus. Words that regularly occur together therefore have similar vectors. In the next stage, the matrix is transformed and subjected to singular value decomposition, a process that decomposes it into independent principal components. Patterns of word co-occurrence can then be captured by considering the set of components (typically around 300) that account for the greatest amount of variance in the data. This process reduces the dimensionality of the data while extracting the “latent” statistical structure underlying patterns of contextual co-occurrence. Following this process, each word is associated with a shorter vector that can be thought of geometrically as representing its position in a high-dimensional space. Words that occur in similar documents to one another occupy similar locations in the space. The proximity of two words can be computed, typically by taking the cosine of the angle between them, and this is used as a measure of their semantic relatedness. Semantic representations obtained in this way have been shown to provide a good fit to a range of empirical data on semantic relationships, including relatedness judgements, free association responses and priming effects (Griffiths et al., 2007; Landauer & Dumais, 1997; Rohde et al., 2006).

For the present study, we took the standard computational steps described above but applied these to a rather different set of data. We were interested in patterns of co-occurrence among objects in the environment and not among words in language use; therefore, we required a dataset from which we could compute the frequency with which particular objects appear together in the same environments. We used a subset of SUN2012 scene database (Xiao, Hays, Ehinger, Oliva, & Torralba, 2010), which is a large collection of photographs sampled from a broad range of environments (e.g., indoor, outdoor, domestic, and public locations). 15,875 of these images have been labelled by a single individual using a computerised toolbox (Barriuso & Torralba, 2012). Labelling involved manually identifying each distinct object in the image and giving it a verbal label. Fig. 1 shows lists of object labels for three example images. We treated each of the 15,875 images as a different environment and extracted the list of object labels associated with each. Sometimes a set of spatially contiguous objects were grouped together under a single label (e.g., *trees* in Fig. 1). When a plural label was used in this way, it was impossible to know precisely how many objects were being referred to. We therefore treated these labels as single instances of the object of question and added them to the total for the singular form of the object name. It is also worth noting that labelling is an inherently subjective process and that one can think of many instances where the identification of an object might be ambiguous. For example, some people might refer to the cups in the kitchen image as “mugs”. In practice, this ambiguity was minimised by the fact that all of the images were labelled by the same individual, who was highly consistent in her vocabulary and approach to labelling across the whole corpus of images (Barriuso & Torralba, 2012). For this reason, we did not perform any other editing of the object names in the database. We did, however, exclude from the analysis any objects that appeared in fewer than 10 images, in case the small number of occurrences of these items was not representative of their true environmental distribution. This filtering resulted in a total of 921 unique object names. We used these objects to generate an object-by-image matrix that recorded the frequency with which each object appeared in each image in the database.

Next, we transformed the values in the matrix according to the standard procedure for LSA (Landauer & Dumais, 1997). First, frequency counts were log-transformed to reduce the influence of very high values. Then, the logs associated with each object were multiplied by that object׳s entropy (*H*) in the database as a whole, defined for an object *i* according to the formula

$$H\left(i\right)=1+\sum_{j}\frac{p_{i,j}\;\log\left(p_{i,j}\right)}{\log\left(N\right)}$$

where *j* indexes all of the images in which the object appears, *N* represents the total number of images in the database and *p*_*i,j*_ represents the frequency of object *i* in image *j* divided by the total frequency of object *i* in the database. This transformation weights the matrix such that objects that appear in a wide variety of images have less influence on the resulting representations. We performed singular value decomposition on the transformed matrix, resulting in a representation of each object as a vector that described its location in a high-dimensional space. Objects that appear in similar environments to one another occupy similar locations in the space (i.e., they have similar vectors). Singular value decomposition provides a representation of the data across a large number of orthogonal dimensions, rank ordered in terms of the amount of variance that they account for in the original matrix. Later dimensions explain little variance and are unlikely to contribute meaningful information to the representations, so most applications of LSA discard all dimensions beyond a particular cut-off point. In this study, we set this cut-off at 70 dimensions, based on pilot investigations that varied the number of dimensions systematically. We describe these investigations in more detail in Appendix. Briefly, we attempted to maximise the similarity between the representations derived from LSA and with representations based on a published database of semantic features (McRae et al., 2005, described in the next section). We defined similarity between two objects by the cosine of the angle between their vectors. By computing the vector cosines for all pairs of objects, we constructed a similarity matrix from the data. We refer to this set of similarities as the *scene dataset.* If patterns of object co-occurrence provide information about the semantic relationships among objects, then we would expect this similarity matrix to resemble that obtained from other approaches to semantic representation. The next section describes how we assessed this.

### 2.2 Comparison with other sources of semantic information

We compared the semantic structure among objects obtained from the scene dataset with two established approaches used to obtain information about the structure of object concepts: (1) feature listing and (2) LSA performed on a text corpus. For feature listing, we used the McRae et al. (2005) dataset. These data were obtained from a large cohort of undergraduate students, who generated semantic features for a total of 541 objects. The authors compiled a list of all the features produced across the full set of objects and generated a vector representation for each object based on the number of participants who produced each feature for that object. They generated a similarity matrix by computing cosines between the vectors of the objects. We refer to this data as the *feature dataset.*

For the traditional application of LSA to a text corpus, we used the LSA representations generated by Hoffman, Lambon Ralph, and Rogers (2013) and Hoffman, Rogers, and Lambon Ralph (2011). These authors applied LSA to the text of the British National Corpus. The corpus consists of texts from 3125 separate documents which were sub-divided into shorter “contexts”, each with a length of 1000 words. This resulted in 87,375 contexts containing over 87 million words (tokens). The size of the corpus is typical of that used in most applications of LSA to verbal data; however, it greatly exceeds the size of the scene dataset. This reflects the relative ease of compiling large corpora from written sources, compared with the manual labelling required for each image in the scene dataset. The corpus was subjected to LSA using the same method as the scene dataset (including log entropy transformation and SVD). In total, Hoffman et al. derived semantic representations for 38,456 different words in this way. We refer to these data as the *verbal LSA dataset.* The first 300 dimensions of the resulting vectors were taken as semantic representations for the words, with cosine between vectors taken as the measure of two words׳ semantic relatedness. The use of 300 dimensions was based on comparisons with feature listing data (see Appendix).

**Fig. 2:**
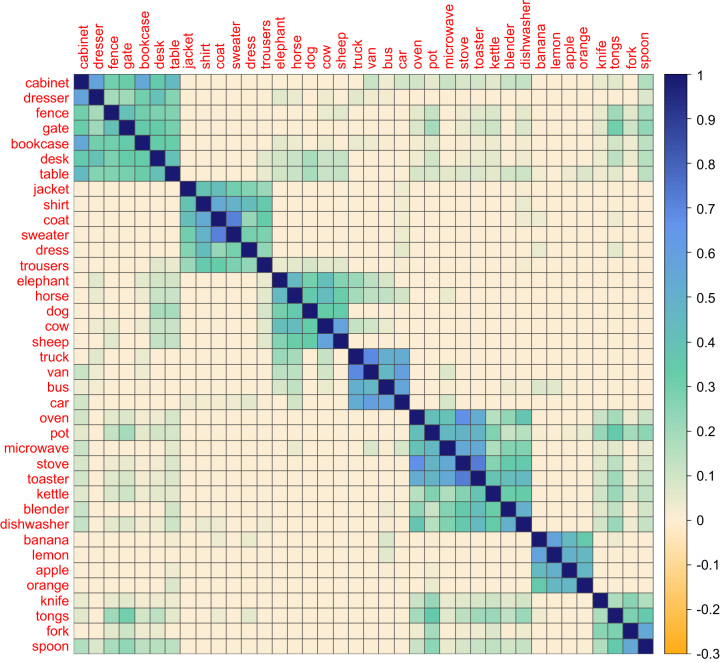
Similarity matrix for a selection of objects in the feature dataset. Colour scale indicates the cosine similarity between pairs of objects (1=identical and 0=no similarity). Objects are ordered according to results of a hierarchical clustering algorithm applied to the data. (For interpretation of the references to colour in this figure legend, the reader is referred to the web version of this article.)

**Fig. 3:**
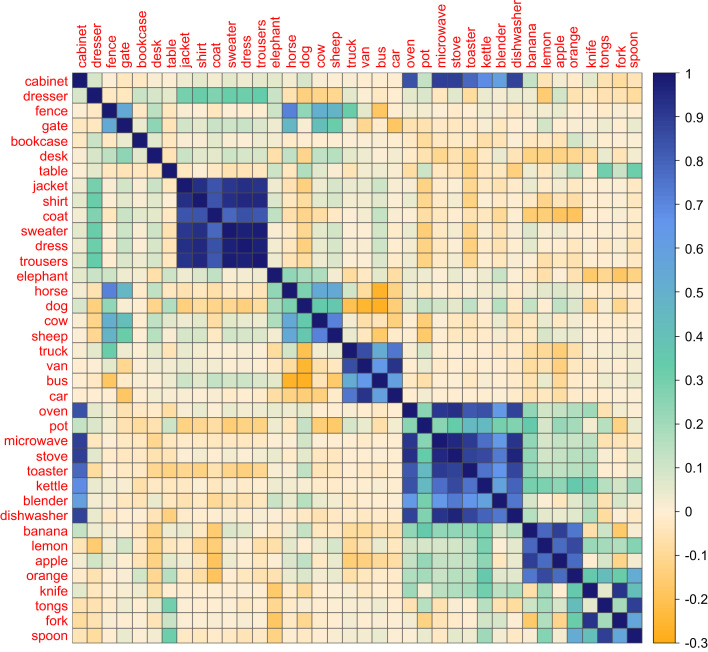
Similarity matrix for a selection of objects in the scene dataset.

**Fig. 4:**
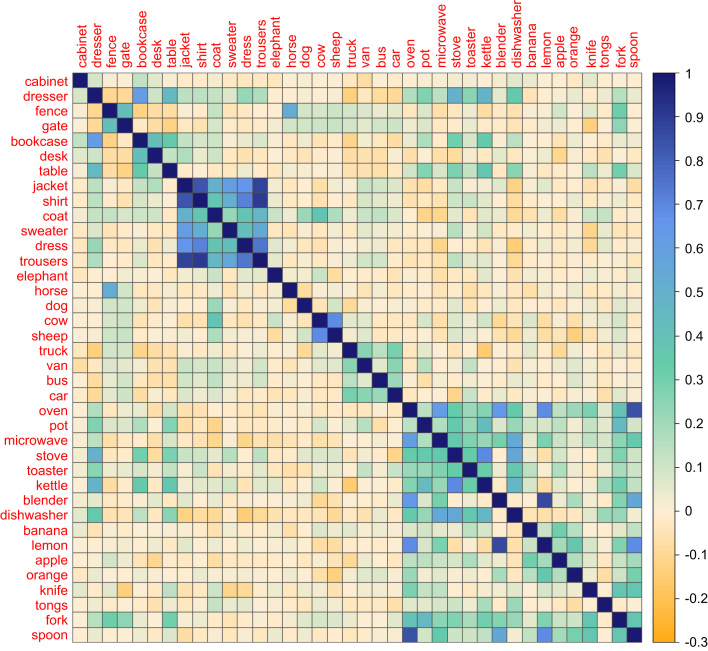
Similarity matrix for a selection of objects in the verbal LSA dataset.

In order to compare the representations from the scene dataset with those of the feature and verbal LSA datasets, we looked for object names that were present in all three sources. There were 122 such objects. We computed similarity matrices for these 122 objects using each set of data and subjected these to statistical analyses described below. To visualise the semantic structure present in each dataset, we manually selected an illustrative subset of 38 objects that spanned a range of semantic categories and constructed matrix plots to represent the structure among these objects. This subset appears in Figs. 2–4.

## 3 Results

### 3.1 Qualitative comparison of the three datasets

Fig. 2 shows semantic feature similarity for 38 objects using data from the McRae et al. (2005) feature listing database. The maximum possible value in this figure is one, indicating that two items have identical features, and the minimum is zero, indicating no shared features (negative values are not possible because items cannot be negatively associated with features). As a number of previous studies have shown (Cree & McRae, 2003; Garrard et al., 2001; Vinson et al., 2003), objects from the same taxonomic category tend to share features. This is apparent in the figure: there are a number of distinct clusters of closely related objects, including animals, fruits, vehicles and clothes. In contrast, most of the between-category similarity values are zero, indicating that objects from different categories generally share no features at all in this dataset. While the object groupings generally form intuitive categories, there are occasional outlier items. For example, the cluster in the top-left corner of Fig. 1 consists mainly of items of furniture but also contains *fence* and *gate*. At one level, this grouping is understandable because they do have some properties in common: all of these items are man-made structures, often made of wood and assembled using screws and nails. However, fences and gates have rather different functions to items of furniture and are found in different environments. The feature dataset appears to be insensitive to these important differences.

Fig. 3 shows similarity between the same objects based on our novel application of LSA to the scene dataset. The objects are arranged in the same order as shown in Fig. 2 to aid comparison. Here, a value of one indicates two items with identical LSA vectors, indicating that they occur in identical environments, with smaller positive values indicating weaker similarities in environmental occurrence. Values close to zero (including negative values) indicate item pairs that occur in unrelated environments. Much of the category-level structure observed in the feature dataset is also present in the scene dataset. Similarities are particularly strong amongst items of clothing, vehicles, kitchen appliances and fruits. This provides initial support for our hypothesis that semantically related objects tend to co-occur in the same or similar environments. One notable exception is the items of furniture, which do not share much similarity. This presumably occurs because different types of furniture, despite sharing basic properties, are found in different environments (desks in offices, dressers in bedrooms etc.). For the same reason, the scene dataset identifies *fence* and *gate* as similar to one another but distinct from the items of furniture. There are also some patterns of similarity that cross taxonomic category boundaries and instead reflect associative relationships between items. For example, *dresser* is identified as somewhat related to all of the items of clothing. The cooking appliances are strongly related to *cabinet* and somewhat related to the fruits, presumably because all of these items frequently co-occur in kitchens. Finally, animals that are found on farms are related to *fence* and *gate* (but *elephant* is not). In general, it seems that semantic relationships based on object co-occurrence reveal both within-category and between-category relationships.

Object similarities derived from the typical application of LSA to linguistic data (Hoffman et al., 2013) are presented in Fig. 4. While there are some sets of strongly related objects (e.g., items of clothing; vehicles), the semantic structure in this dataset appears less coherent than the other two. Like the scene dataset, similarities in the verbal dataset reflect a mixture of categorical and associative relationships. The fruits are identified as somewhat related to many of the kitchen appliances and utensils, for example. In this dataset, *fence* and *gate* are correctly identified as distinct from items of furniture but are both somewhat related to *fork*. This may indicate a lexical association with another sense of the word *fork* (i.e., a fork in the road). In all, however, the verbal dataset appears to provide a less coherent picture of the semantic relationships among objects, despite being based on a much larger corpus of source data. The feature dataset and scene dataset also appear to converge more closely with one another than the verbal LSA dataset. We test these suppositions next.

### 3.2 Statistical comparison of semantic structure in each dataset

Next, we assessed formally the degree of convergence between semantic representations derived from the feature dataset, the scene dataset and the verbal dataset. We computed similarity matrices for each of the three datasets, this time using all 122 objects that were present in all three datasets. We then computed the degree of correlation between the values in each pair of matrices. This method is commonly used to compare similarity matrices obtained from different sources (Devereux et al., 2013; Dilkina & Lambon Ralph, 2012; Peelen & Caramazza, 2012). A positive correlation between two matrices indicates that they contain similar information about the relationship between items (i.e., pairs of items that have high similarity values in one matrix tend to have high similarity values in the other). The correlations between our three datasets are presented in Table 1. All are significantly positive, indicating that they converge to some degree in giving similar conclusions regarding the organisation of object concepts.^1^ Feature listing is the most commonly used method to elucidate semantic structure and, as we have already seen, it provides a clear and categorical structure. It is therefore a useful standard against which to compare the two LSA-based sets of results. The scene dataset has a stronger correlation with the feature dataset than the verbal LSA dataset (*z*=4.05, *p*<0.001), indicating the structure derived from our novel analysis of object co-occurrence more closely resembles feature-derived semantic structure than does the structure obtained from the typical application of LSA to lexical co-occurrence. As a further test of this, we performed a multiple regression analysis in which we used the scene and verbal LSA similarity matrices as simultaneous predictors of the feature similarity matrix. The results are shown in Table 2. Both datasets made significant independent contributions to the model, suggesting that each provides unique information about the organisation of object knowledge. Nevertheless, the standardised regression coefficients (*β*) indicate that the scene dataset is the stronger predictor, indicating that the semantic structure obtained through analysis of object co-occurrence is more closely related to feature-based semantic representations.

### 3.3 Relationship of co-occurrence based datasets to specific types of feature

In a final analysis, we investigated whether the semantic structures derived from verbal and scene analysis were related to a particular class of semantic feature. Features produced in feature generation studies are often classified as perceptual (properties observed through the senses), functional (properties relating to how an object is used) or encyclopaedic (other properties, often acquired verbally). Most studies combine all feature types together when analysing the organisation of concepts, as we have done up to this point. However, different forms of organisation can be revealed if features of each type are analysed separately (Cree & McRae, 2003; Dilkina & Lambon Ralph, 2012). To test how each feature type relates to semantic structure based on co-occurrence, we generated three separate similarity matrices from the feature dataset: one based only on perceptual features, one on functional features and one on encyclopaedic features. We then computed the correlation between each of these matrices and the matrices based on the scene and verbal LSA datasets. The results are shown in Table 3. The structure derived from the scene dataset was notably more strongly correlated with semantic structure obtained from encyclopaedic features than with the structure derived from either perceptual (*z*=7.41, *p*<0.001) or functional (*z*=6.48, *p*<0.001) features. Closer inspection of the encyclopaedic features revealed that these features sometimes specified the environment in which objects were typically located (e.g., *found in kitchens*). It therefore appears that the scene dataset is systematically capturing information that is sometimes specified spontaneously by participants when they are asked to generate object features. The structure generated by the verbal LSA analysis was also more strongly correlated with encyclopaedic feature structure than either perceptual (*z*=4.76, *p*<0.001) or functional (*z*=2.18, *p*=0.03) structure. The explanation for this is unclear, though it may reflect the fact that encyclopaedic knowledge is often expressed verbally and is therefore strongly represented in the verbal corpus. At the same time, it is interesting to note that the verbal dataset was positively correlated, albeit weakly, with the perceptual feature structure, which suggests that perceptual experience may be coded indirectly in the verbal corpus (in verbal descriptions of objects or scenes, for example).

## 4 Discussion

The structure of semantic relationships amongst concepts is a key topic in cognitive neuroscience, with two influential approaches used to infer such relationships. The first assumes that object concepts are related to the degree to which they share basic properties or features (Collins & Quillian, 1969; McRae et al., 1997; Rogers et al., 2004; Rogers & McClelland, 2004; Tyler et al., 2000; Vigliocco et al., 2004). The second assumes that concepts are related to the degree that the words that refer to them occur in similar linguistic contexts (Firth, 1957; Griffiths et al., 2007; Landauer & Dumais, 1997; Lund & Burgess, 1996; Rohde et al., 2006). Here, we investigated whether the second approach could be applied to non-verbal patterns of object co-occurrence in natural environments. We used latent semantic analysis (LSA; Landauer & Dumais, 1997) to derive representations for object concepts based on their distribution over a corpus of labelled photographs of scenes. The resulting representations coded objects as related based on the degree to which they appeared in similar scenes. Overall, there was considerable association between the relationships revealed through this analysis and those derived from the established approaches of feature similarity analysis and distributional analysis of linguistic data. We found that, like the feature similarity approach, representations derived from the scene dataset revealed strong relationships among category co-ordinates. However, unlike the feature approach, analysis of the scene dataset also captured information about cross-category associations. For example, oranges and lemons had similar representations to one another but also shared some representational overlap with knives. Overall, LSA applied to the scene dataset provided a closer fit to the feature-based semantic structure than LSA applied to a linguistic corpus, though both appeared to provide complementary information.

The major contribution of this work is to demonstrate that the distributional principle – the idea that concept co-occurrence is an important source of information about the relationships between concepts – can be successfully extended from the language domain to non-verbal visual experience. Environmental co-occurrence is a ubiquitous element of everyday experience. We rarely perceive objects in isolation; instead, individual objects are embedded in a variety of often complex environments. The present work demonstrates that considerable statistical regularities are present in the distribution of objects across environments and that these can be extracted to provide meaningful information about the conceptual significance of the objects themselves. In parallel, the distribution of words over linguistic contexts provides data about their semantic relationships and semantic features provide insights into the basic perceptual properties of objects and the functions for which we use them. Conceptual representation likely emerges as the result of merging all of these aspects of experience. Discovering the optimum method for combining this diverse database is a major challenge, though recent studies have made progress in integrating feature knowledge with lexical co-occurrence patterns (Andrews et al., 2009; Durda et al., 2009; Johns & Jones, 2012; Steyvers, 2010). Our results suggest that inclusion of environmental object co-occurrence information could improve such models further. In addition, using object co-occurrence statistics may prove an effective method for classifying images, with potential applications for coding semantically similarity amongst images.

There is also considerable interest in representational structure in the field of neuroimaging, with many researchers using multi-voxel pattern analyses in an attempt to discover the structure of object representations in the brain (Devereux et al., 2013; Fairhall & Caramazza, 2013; Kriegeskorte et al., 2008; Peelen & Caramazza, 2012). These approaches often start from the assumption that object representations are organised by category and use this principle to guide their analyses. Indeed, for many years researchers have investigated category-level distinctions in occipitotemporal regions involved in visual object recognition (Kanwisher, 2010; Martin, 2007). However, at a higher conceptual level, cross-category semantic associations are likely to also be important. It is interesting to note that a recent study found that voxels in the anterior temporal cortex, known to be a key site for conceptual representation, distinguished between objects typically found in kitchens and those found in garages (Peelen & Caramazza, 2012). More detailed information on environmental co-occurrence may therefore prove useful in interpreting the data now emerging from these sophisticated neuroimaging paradigms.

Finally, we note some potential limitations to our approach. The first is that the corpus we used to explore object co-occurrence is much smaller than those used to investigate lexical co-occurrence. The scene corpus contained a little over 270,000 labelled objects. In contrast, linguistic corpora typically contain many millions of words: the verbal corpus used in this study comprised 87 million lexical tokens. With this in mind, it is perhaps surprising that the scene dataset showed a greater degree of convergence with the feature list data than the verbal corpus did. This may indicate that there is a higher level of regularity in environmental object co-occurrence than there is in the lexical co-occurrences of the words that refer to them, with the result that meaningful structure can be extracted from a much smaller sample. Alternatively, it is possible that the feature listing data and scene dataset were primarily sensitive to broad category-level groupings while the larger verbal dataset captured fine-grained distinctions between individual items not present in the other datasets. This possibility could be explored in future studies by investigating object relations at different levels of specificity.

The scene dataset was based on a smaller corpus because it relied on laborious manual identification and labelling of the objects in each scene. This process brings with it other challenges. Object identification and labelling is a complex cognitive task with considerable scope for individual variation. Many objects are associated with multiple near-synonymous labels (e.g., *cups* vs. *mugs*) or can be labelled at different levels of specificity (e.g., *dog* vs. *poodle*). Multi-component objects can be broken down into more basic constituent parts (e.g., a *car* could theoretically be sub-labelled with *wheels, lights, mirrors, windscreen* etc.). We minimised these sources of variation by using a set of images that were all labelled by a single individual who was reported to be highly consistent in her vocabulary and approach to labelling (Barriuso & Torralba, 2012). A more representative picture could emerge from sampling from a larger group of individuals, though this would entail consideration of whether and how to standardise labels across participants. Similar issues are encountered in feature listing studies, where a particular property can be expressed in variety of different ways (e.g., “a lion is dangerous” vs. “a lion can kill people”; Garrard et al., 2001). Another issue for future consideration is the fact that the same label can be applied to a variety of objects which differ to some degree in their perceptual and functional characteristics. Some of this variation is likely to be systematic with respect to environment. For example, the type of chair typically found in an office is different to that found in a living room and both differ from the chairs found in a classroom. Any method that classifies objects with verbal labels (including feature databases and lexical co-occurrence analyses) is insensitive to these variations. More generally, in natural language we often group collections of items under superordinate labels rather than describing each individually. For example, we are more likely to say “a bowl of fruit” than “a bowl of apples, oranges and bananas”, or we might describe an untidy room as “a tip” rather than explaining exactly which items were out of place. For this reason, information about the composition of environments may be under-represented in linguistic corpora. In contrast, in the scene dataset this information is represented explicitly.

An alternative approach to labelling may be to incorporate image-based information more directly into representational models. Computer vision researchers often represent images as constellations of low-level visual “features” extracted from the pixels of the images using automated algorithms (e.g., Yang, Jiang, Hauptmann, & Ngo, 2007). These methods use similar statistical techniques as those used in distributional analyses of text corpora, raising the possibility of models that integrate visual and text-based information. Bruni, Tran, and Baroni (2011) have recently taken an important step in this direction by separately deriving representations from lexical co-occurrence and automated image analysis and then combining these in a single representational space. They found that the lexical and visual sources provided complimentary information about the objects, with the best fit to empirical data occurring when both were combined. This method is somewhat different to the one we have taken here; however, it highlights the exciting prospect for future representational models that combine elements of perceptual and verbal experience. This could lead to better understanding of the true multi-modal nature of object concepts and, in turn, their neural basis.

## Figures and Tables

**Fig. 5:**
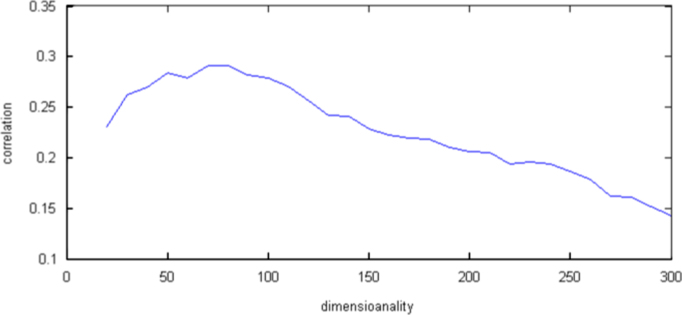
Correlation between the similarity matrix derived from the feature dataset and matrices derived from the scene dataset using varying numbers of dimensions.

**Table 1:** Correlations between similarity matrices derived from each dataset.

	Feature dataset	Scene dataset	Verbal LSA dataset
Feature dataset	1		
Scene dataset	0.29⁎	1	
Verbal LSA dataset	0.23⁎	0.30⁎	1

⁎ *p*<0.001.

**Table 2:** Results of multiple regression analysis predicting feature dataset similarities from the other two datasets.

	*B*	Standard error	*β*	*t*
Scene dataset	0.13	0.006	0.25	21.4⁎
Verbal LSA dataset	0.11	0.008	0.16	13.8⁎

⁎ *p*<0.001.

**Table 3:** Correlations of similarity matrices for scene and verbal LSA datasets with similarity matrices generated from each type of semantic feature separately.

Feature type	Scene dataset	Verbal LSA dataset
Perceptual	0.18⁎	0.14⁎
Functional	0.20⁎	0.19⁎
Encyclopaedic	0.30⁎	0.21⁎

⁎ *p*<0.001.

## Appendix A Selecting number of dimensions for the scene dataset

An important issue when performing LSA is the selecting an appropriate number of dimensions to contribute to the representation. The dimensions returned by singular value decomposition are ordered by the amount of variance they explain in the data. Early dimensions explain large amounts of variance, indicating that they capture reliable and significant trends within the dataset. Later dimensions explain less variance, until eventually a point is reached where further dimensions may simply “fit the noise” rather than extracting stable patterns from the data. Applications of LSA therefore require investigators to select an optimum number of dimensions for their data. This is often determined by reference to an empirical standard. For example, Landauer and Dumais (1997) generated semantic representations using varying numbers of dimensions and tested the ability of these representations to discriminate between words in a multiple-choice vocabulary test. They observed that performance followed an inverted U-shape. Models with a small number of dimensions (<50) performed poorly on the test, presumably because they excluded dimensions that coded important information about the semantic organisation of the words. Equally, models with more than 1000 dimensions also performed poorly, presumably because these included dimensions that captured little meaningful variation in the data and instead contributed noise to the representations. Between these extremes, optimum performance was achieved by models that used around 300 dimensions. This threshold is commonly used in applications of LSA to linguistic data (e.g., Hoffman et al., 2013; Rohde et al., 2014).

We used a similar approach to determine the optimum number of dimensions for our scene dataset. As our empirical standard, we used the matrix of similarities between objects derived from a semantic feature database (McRae et al., 2005; see main text for further details). We used the LSA results for the scene dataset to generate a series of similarity matrices for same objects, varying the number of dimensions from 20 to 300 (in steps of 10). We then computed the correlation between each of these matrices and the feature-based matrix. A positive correlation between two matrices indicates association in the information each contains, with stronger correlations implying greater correspondence. The results are shown in Fig. 5. Similar to the results of Landauer and Dumais (1997), the distribution of correlations followed an inverted U-shape. Models with very few or very many dimensions exhibited the weakest correlations with the feature dataset. The peak correlation value was obtained for the model that used 70 dimensions. We used this model for all analyses in the paper, on the basis that it provided the best fit to the empirical semantic feature dataset. It is worth noting that 70 dimensions is somewhat lower than the number of dimensions typically used in LSA. This is most likely a consequence of the small size of the scene dataset, relative to linguistic corpora used in standard applications of LSA.

Finally, we repeated this analysis for the verbal LSA data (from Hoffman et al. (2013)) that we compared with the scene dataset. Again, we varied the number of dimensions included in the representations between 20 and 300 and assessed the degree of correlation with the feature dataset. In this case, the peak correlation occurred for 300 dimensions, in line with other applications of LSA to linguistic data.

## Footnotes

1 It is important to note we determined the number of dimensions to use in both LSA analyses (scene dataset and verbal dataset) by maximising the correlation of their similarity matrices with the matrix derived from feature listing (see Appendix for details). Therefore, the results reported here represent the maximum correlations with feature listing that were obtained across a range of dimension values.
